# Life-long follow-up of second primary lung and extra-pulmonary cancer in lung cancer patients is needed

**DOI:** 10.7150/jca.44581

**Published:** 2020-05-22

**Authors:** Bingqing Ma, Guoyou Qin, Yue Zhang, Chang Su, Zhenyu Wu

**Affiliations:** 1 Department of Biostatistics, School of Public Health, Key Laboratory of Public Health Safety and Collaborative Innovation Center of Social Risks Governance in Health, Fudan University, Shanghai, China.; 2 National Institute for Nutrition and Health, Chinese Center for Disease Control and Prevention, China.

**Keywords:** lung cancer, second primary cancer, competing risk, nomogram, follow-up

## Abstract

**Background:** Lung cancer (LC) patients are at high risk of developing second primary cancer (SPC). This study aimed to explore the risk factors associated with SPC and provide an individualized risk prediction model for LC patients.

**Methods:** Initial primary lung cancer (IPLC) patients diagnosed between 1998 and 2011 were identified from the Surveillance, Epidemiology, and End Results (SEER) database. A Fine-Gray multivariate competing-risk model was used to estimate the risk of SPC, and the model was assessed regarding discrimination and calibration. A nomogram was designed for clinical convenience to predict the 3-, 5-, and 10- year probabilities of developing SPCs.

**Results:** A total of 142,491 IPLC patients were considered in this study and 14,374(10.01%) developed SPC within a maximum study period of approximately 19 years. Seven independent prognostic factors were identified according to the competing-risk model, and the SEER summary stage and surgery were the strongest predictors. The model was well calibrated and had good discrimination ability(*C*-index = 0.746).

**Conclusions:** LC survivors had an increased risk of SPC and factors associated with good prognosis often predicted SPC. Consideration should be given to increasing the duration of routine follow-up even after 10 years of initial diagnosis for those at the highest risk and site-specific follow-up strategy is also required.

## Introduction

Lung cancer (LC) is the most commonly diagnosed cancer and the leading cause of cancer-related deaths in the United States. The latest estimates show that there would be more than 20,000 incident LC cases and 14,000 deaths due to LC in 2019 1. As a result of the widespread adoption of advanced diagnostic and therapeutic techniques in clinical practice, the survival time has significantly increased among LC patients during the past decades. However, compared to the general population, those survivors remained at high risk of developing a second primary cancer (SPC) 2, and the threat might persist for more than 10 years after the initial diagnoses 3.

Previous studies have reported that age 4,5, white race 6, adenocarcinoma 7, surgical treatment 8, alcohol consumption 9 and tobacco use 10 were associated with the development of SPCs. However, those studies had either inherent selection biases due to their limited sample sizes 11-13, or estimating biases due to improper statistical approaches to the evaluation of the risk of developing SPC 2. In addition, several studies only considered this problem from the perspective of a single variable rather than incorporating multiple factors simultaneously 2,7. Furthermore, in studies concerning only second primary lung cancer, the overall profile of SPCs after IPLC, especially extra-pulmonary tumors, remained unknown 5.

Prediction of SPCs based on patients' individual characteristics not only provides the opportunity for informed decision-making but also promotes patient adherence to medication through effective doctor-patient communication. In the current study, we aimed to develop a multivariate competing-risk prognostic model to predict the risk of SPCs for LC patients. In addition, for clinical convenience, we designed a nomogram, a pictorial representation of the above-mentioned competing-risk model.

## Materials and methods

### Study population

The Surveillance, Epidemiology, and End Results (SEER) is an authoritative source for cancer patients. Data regarding IPLC patients were extracted from the SEER 18 database (April 2019). Patients were considered qualified if their 1) reports were not from death certificates or autopsy, 2) tumor information was correctly recorded, and 3) age was between 18 and 79 years. We subsequently excluded the SPC cases diagnosed within 2 months after the initial diagnosis, and patients with missing values on important variables. Although the SEER April 2019 submission involved cases resisted by the end of 2016, we limited the analysis to patients diagnosed between 1998 and 2011 to ensure at least 5 years of follow-up after the initial diagnosis. The enrolled patients were divided into two cohorts depending on their SPC status. A study flowchart is presented in supplemental Figure S1.

### Outcome and covariates

The primary outcome was the diagnosis of SPC 2 months after the initial diagnosis. The SEER rules for defining an SPC depend on the site of origin of cancer, date of diagnosis, histological classification, tumor behavior (in situ vs. invasive), and laterality of paired organs 14. In general, all cancers occurring more than 2 months after the initial diagnosis were considered as separate primary cancer unless the medical records stated that they were recurrent or metastatic 15.

Potential covariates were pre-specified based on the literature and availability in clinical practice. We collected data regarding demographic variables such as sex, race, and the age at diagnosis of IPLC and tumor characteristics such as the size, stage, grade, histological classification, laterality and surgical treatment. Histological classification was performed according to the International Classification of Diseases for Oncology, 3rd edition. Tumor stage at diagnosis was defined using the SEER summary staging system, including localized, regional, or distant disease.

### Statistical analyses

The demographic and clinical characteristics of patients were summarized with descriptive statistics and compared with Chi-square tests. In the study of SPC, it is noteworthy that a considerable proportion of patients might die before SPC. Using standard Kaplan-Meier or Cox regression methods without considering deaths as a competing event would lead to an overestimated risk of developing SPCs 16. Thus, a Fine and Gray subdistribution hazards model 17, was constructed to estimate the risk of SPC conditioned on the covariates of interest, and a death before the diagnoses of SPCs was considered as a competing event. Stepwise backward elimination method was used to select variables to be included in the final prediction model. A variable was considered for addition to or subtraction from the set of variables based on the BICcr 18.

To facilitate usage among researchers and clinicians, a competing-risk nomogram was proposed. The sum of the independent factors was calculated to estimate the 3-year, 5-year and 10-year probabilities of developing an SPC. Additionally, patients could be stratified into three risk-groups according to the quantiles of the total points: the high (>3^rd^ quantile), moderate (1^st^ to 3^rd^ quantile) and low (<1^st^ quantile) risk-group. Validation of the proposed nomogram was evaluated with respect to calibration, which was performed using 200 bootstrap resamples, and the concordance index (C-index), which measured the classification accuracy. All analyses were performed using R (http://www.r-project.org), “crrstep” and “cmprsk” packages were used for statistical modelling and “rms” package was used for plotting the nomogram. A two-side *P*-value of <0.05 was considered statistically significant.

## Results

### Characteristics of study population

A total of 142,491 LC patients were identified in the current study, and 14,374 (10.1%) of those developed an SPC within the median and maximum follow-up time of 10.6 and 18.9 years, respectively. Of the enrolled study patients, 77,314 (54.3%) were male; most were white (82.2%) and older than 55 years (84.8%). Adenocarcinoma and squamous cell carcinoma were the two most common histologic types, accounting for 44.1% and 27%, respectively. IPLC patients with tumor size larger than 4cm accounted for almost half of the patients without SPCs while the proportion was only a quarter among patients with SPCs. The proportion of patients with localized disease was the highest among those patients who developed SPCs (56.7%) and the lowest (26.7%) among those without SPCs compared with regional and distant disease. More than half of the number of patients (54.2%) without SPCs and 14% of patients with SPCs did not undergo surgery during the treatment of IPLC (Table 1).

**Figure 1:**
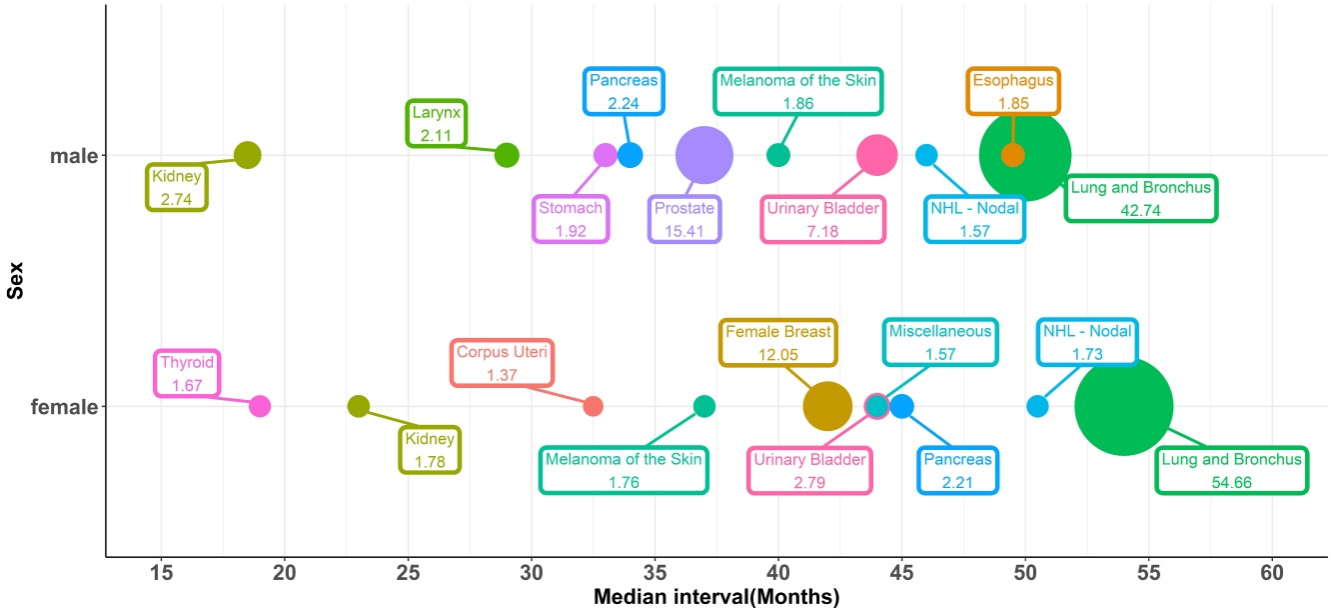
Bubble plots for the top ten most vulnerable sites of SPCs and median intervals between the initial diagnosis and the development of SPCs, stratified by sex.

The top ten most vulnerable sites of SPCs and median intervals, which means the time between the initial diagnosis of IPLC and the development of SPCs, were shown in Figure 1. Lung cancer was the most commonly diagnosed SPC, representing 54.7% and 42.8% of all SPCs in female and male patients, respectively. 15.4% of the male SPC was prostate cancer, and 12% of the female SPC was breast cancer. Both urinary bladder cancer and kidney cancer were vulnerable sites of SPCs. For female patients, the median interval of SPC occurrence was 54, 42, 44, and 23 months for lung cancer, female breast cancer, urinary bladder cancer, and kidney cancer, respectively. For males, the median interval was 50, 37, 44, and 18.5 for lung cancer, prostate cancer, urinary bladder cancer, and kidney cancer, respectively.

### Fine-Gray multivariate competing-risk model and evaluation

Variables listed in Table 1 were involved in a multivariate competing-risk model and the most important variables were selected based on the BICcr. The retained variables, which were significantly associated with the development of SPC, included sex, age at diagnosis, race, tumor size, SEER summary stage, histology, and surgery. The subdistribution hazard ratio (SHR) for each predictive factor is shown in Table 2. Surgery emerged as the strongest predictor, compared with those who did not have surgery; patients treated with lobectomy were most susceptible, followed by sublobectomy and then pneumonectomy. Our proposed model showed relatively good discrimination and calibration, because the optimism-corrected *C*-index was 0.746 and the dots on the calibration plots, which represented the observed probability against the predicted probability, were close to a 45° diagonal line (Supplementary file, Figure S2).

### Nomogram

**Figure 2:**
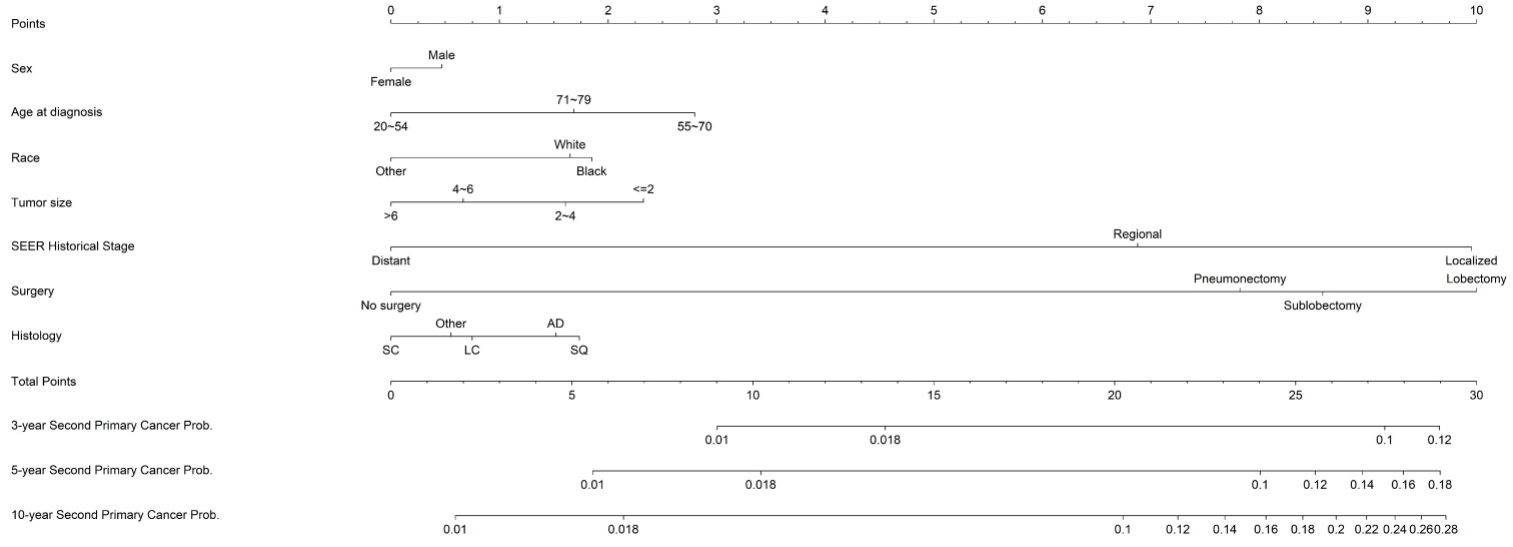
Nomogram estimating the individual-level 3-, 5- and 10- year probabilities of SPC among IPLC patients. To obtain the predicted 3-, 5- and 10-year probability of developing SPC, firstly, draw a vertical line to the “Points” scale for each predictor according to patient individualized traits; Secondly, sum the points for all predictors and locate the sum on the “Total Points” scale; thirdly, draw a vertical line towards axes at bottom to determine the 3-, 5- and 10- year probabilities respectively. Additionally, patients could be categorized as high-risk (>=24), moderate-risk (7~24) and low-risk (<7) group according to the total points.

Based on the optimal model with the significant predictors, a prognostic competing-risk nomogram was constructed for clinical convenience (Figure 2). SEER summary stage, age, tumor size, and surgery emerged as the strongest predictors and contributed most to the discrimination. The scores of each prognostic factor used for plotting the nomogram were also provided (supplemental Table S1). The 3-, 5- and 10-year probability of developing SPCs could be estimated by adding the points of each predictor. The 1^st^ and 3^rd^ quantiles of the summarized total points were 7 and 24; thus, patients could be categorized into high-risk (≥24), moderate-risk (7~24) and low-risk groups.

## Discussion

A large retrospective cohort study showed that almost one in ten cancer patients developed SPC, and 55% of SPC patients died due to SPCs rather than the initial cancers 19. However, the current guidelines pay insufficient attention to SPC in LC patients. The lack of guidance on what constitutes high-risk SPC patients is one factor that has contributed to variations in the follow up of LC patients. For the prediction of the risk of SPC in LC patients, previous studies usually only consider second primary lung cancer, while extra-pulmonary tumors were excluded 5. In fact, more than half of SPC cases in LC patients were actually extra-pulmonary malignancies. To our knowledge, this was the largest study to construct a model to simultaneously predict the risk of both second primary lung cancer and second primary extra-pulmonary cancer for LC patients.

Our study had the following strengths: first, the findings were made based the SEER program, in which high-quality population data were collected and maintained to avoid the selection bias imposed by a single-center study or small-sample study. Second, the length of the study period was nearly 20 years, which made the findings more robust. Third, competing-risk proportional hazard regression, which accounted for death before the development of SPC as a competing event, was used to obtain unbiased estimates of the risk factors of SPC. Finally, our proposed nomogram, which showed good discrimination and calibration, could be easily used to quantitatively depict the individualized predictive risk of SPC by integrating diverse covariates and to identify those at high risk of developing a subsequent malignancy.

According to our results, a total of 10% of the LC patients developed SPC within a maximum follow-up period of 18.9 years, and the estimated 3-, 5-, and 10-year cumulative risks of SPC for the entire study cohort were 4.2%, 6.4%, and 10.0%, respectively. Several interesting findings deserve mention in the current study. Firstly, when the age at initial diagnosis was considered, a bell-shaped association was observed. The old (age 71-79 years) and young (age 20-54 years) subgroups showed the lowest risk, while middle aged (age 55~70 years) patients showed the highest risk. This phenomenon could be explained by the fact that patients with disease onset at a younger age tended to present with more aggressive disease at diagnosis, leading to an extremely poor prognosis 20. Similarly, old patients tended to have worse physical conditions and more complications 21 and were likely to be with a poor prognosis in comparison with their younger counterparts 22.

Secondly, other acknowledged good prognostic factors, such as early tumor stage, female gender, smaller tumor size, or non-small cell lung cancer (NSCLC) histology, were positively associated with higher prevalence of SPC 23.However, the major reason was the longer survival, and thus, the extended risk period. Other explanations have been proposed: the high incidence of SPC may be related to some specific genotoxic injury. For example, mutations in the tumor suppressor gene p53 and the proto-oncogene K-*ras* were more frequently found in female LC patients than in males 24. However, a previous study found a high frequency of K-*ras* codon 12 mutations in patients at high risk for second primary lung cancer 25.

Thirdly, the long-term effect of treatment on SPC is a major concern in relevant studies and surgery was found the second most important predictor in the current study. Patients treated with surgery have a higher risk of SPC than those who did not, as observed in previous studies. In a single-center study of 1294 patients with early-stage NSCLC who underwent resection, the risk of SPC development ranged from 3%-6% per person-year and did not decline over time 12. Another single-institution study of 947 patients also demonstrated that patients who underwent surgical resection of IPLC were at risk for the development of SPC and the potential risk persisted for ≥5 years after the initial surgery for IPLC 8. Surgical trauma induces systemic inflammation via the release of inflammatory cytokines 26 and such inflammation may accelerate the proliferation of dormant tumor cells and play a role in determining cancer susceptibility. Receiving postoperative radiotherapy may further increase the risk of SPCs 27.

Finally, the result of our study may have some implications for clinical health-care management. The goals of follow-up and surveillance programs for IPLC survivors should not only focus on the detection of recurrence or metastasis, but also on the early detection of a subsequent new primary cancer. Guidelines proposed by major organizations in Europe and North America have consistently recommended intensive follow-up during the first 2 years after surgery 28-30. However, according to our study, the risk of SPCs persist even after 10 years of initial diagnosis. We recommend intensive follow-up for the high-risk survivors. The early identification of SPCs allows for alternative approaches, thereby reducing unnecessary treatments, morbidities, and costs 31. Site-specific follow-up strategy should be proposed, as the top five sites account for about 70% of all SPCs; for example, mammography screening should be considered in female patients. Moreover, cancer survivors may experience more psychological problems after their second diagnosis of cancer 32,33. Depression is one of the major risk factors for non-adherence to cancer therapy; thus, the evaluation of quality of life, depressive symptoms and cancer-specific stress would be recommended during the follow up.

Our study also has limitations. First, data regarding several important risk factors relevant to SPCs, such as smoking status 34,35 , family history of malignancies 36, and body weight 37, were not available from the SEER database, and thus could not be controlled when predicting the individual risk of developing SPCs. As our study cohorts were retrospectively collected within a long period, it is likely the risk of SPC was influenced by the secular trends of anticancer therapies. Improved screening and treatment strategies may have greatly prolonged patients' survival and thus lead to higher risk of exposure to a subsequent cancer 38. Besides, the validity of our proposed nomogram needs further exploration in other population-based datasets.

## Conclusions

In conclusion, LC patients remained at a high risk for the development of SPCs, and the cumulative risk increased over time. According to our multivariate competing-risks analysis, 55-70 years old black men with localized disease, smaller tumor size, and histological diagnosis of squamous cell carcinoma, and who underwent surgical treatment seemed most vulnerable to subsequent cancers. Moreover, a user-friendly competing-risks nomogram was created. Future studies are needed to provide more specific surveillance and health-care strategies for both the physical and psychological health of those survivors.

## Supplementary Material

Supplementary figures.

## Figures and Tables

**Table 1:** Characteristics of the lung cancer patients with and without second primary cancers (SPCs)

Characteristics	Study cohorts, *n* (%)		
	All	Without SPCs	With SPCs
*N* (%)	142491	128117(89.9)	14374(10.1)
**Sex**			
Female	65177 (45.7)	58360 (45.6)	6817 (47.4)
Male	77314 (54.3)	69757 (54.4)	7557 (52.6)
**Age at diagnosis, years**			
20~54	21699 (15.2)	20124 (15.7)	1575 (11.0)
55~70	74682 (52.4)	66385 (51.8)	8297 (57.7)
71~79	46110 (32.4)	41608 (32.5)	4502 (31.3)
**Race**			
Black	16127 (11.3)	14745 (11.5)	1382 ( 9.6)
White	117196 (82.2)	104935 (81.9)	12261 (85.3)
Other	9168 ( 6.4)	8437 ( 6.6)	731 ( 5.1)
**Grade**			
I	12335 ( 8.7)	10391 ( 8.1)	1944 (13.5)
II	43585 (30.6)	37852 (29.5)	5733 (39.9)
III	71581 (50.2)	65657 (51.2)	5924 (41.2)
IV	14990 (10.5)	14217 (11.1)	773 ( 5.4)
**Tumor size, cm**			
≤2	30318 (21.3)	25560 (20.0)	4758 (33.1)
2~4	53511 (37.6)	47466 (37.0)	6045 (42.1)
4~6	31134 (21.8)	28930 (22.6)	2204 (15.3)
>6	27528 (19.3)	26161 (20.4)	1367 ( 9.5)
**SEER summary stage**			
Localized	42315 (29.7)	34165 (26.7)	8150 (56.7)
Regional	51164 (35.9)	45956 (35.9)	5208 (36.2)
Distant	49012 (34.4)	47996 (37.5)	1016 ( 7.1)
**Histology**			
AD	62792 (44.1)	55186 (43.1)	7606 (52.9)
LC	6962 ( 4.9)	6354 ( 5.0)	608 ( 4.2)
SC	12962 ( 9.1)	12456 ( 9.7)	506 ( 3.5)
SQ	38536 (27.0)	34317 (26.8)	4219 (29.4)
Other	21239 (14.9)	19804 (15.5)	1435 (10.0)
**Surgery**			
No surgery	71401 (50.1)	69395 (54.2)	2006 (14.0)
Lobectomy	54539 (38.3)	44491 (34.7)	10048 (69.9)
Pneumonectomy	5664 ( 4.0)	5009 ( 3.9)	655 ( 4.6)
Sublobectomy	10887 ( 7.6)	9222 ( 7.2)	1665 (11.6)
**Laterality**			
Left	59471 (41.7)	53300 (41.6)	6171 (42.9)
Right	83020 (58.3)	74817 (58.4)	8203 (57.1)

**Table 2:** Significant characteristics associated with the development of second primary cancers (SPCs), estimated by the Fine and Gray multivariate competing-risk model

Characteristics	SHR	95%CI	*P*
**Sex**			
Female	reference		
Male	1.06	1.03-1.10	<0.001
**Age at diagnosis, years**			
20~54	reference		
55~70	1.43	1.35-1.51	<0.001
71~79	1.24	1.17-1.31	<0.001
**Race**			
Other	reference		
White	1.23	1.14-1.33	<0.001
Black	1.27	1.16-1.38	<0.001
**Tumor size (cm)**			
>6	reference		
≤2	1.34	1.26-1.43	<0.001
2~4	1.23	1.15-1.30	<0.001
4~6	1.09	1.02-1.16	0.014
**SEER summary stage**			
Distant	reference		
Localized	3.55	3.29-3.83	<0.001
Regional	2.40	2.23-2.59	<0.001
**Histology**			
SC	reference		
AD	1.21	1.11-1.33	<0.001
LC	1.10	0.98-1.24	0.119
SQ	1.25	1.13-1.37	<0.001
Other	1.07	0.97-1.19	0.179
**Surgery**			
No surgery	reference		
Lobectomy	3.57	3.37-3.79	<0.001
Sublobectomy	2.98	2.77-3.21	<0.001
Pneumonectomy	2.71	2.47-2.97	<0.001

## Abbreviations

LC: lung cancer
SPC: second primary cancer
AD: adenocarcinomas
LC: large cell carcinomas
SC: small cell carcinomas
SQ: squamous cell carcinomas
95% CI: 95% confidence interval
SHR: subdistribution hazard ratio
