# Supplementary material for: Life-long follow-up of second primary lung and extra-pulmonary cancer in lung cancer patients is needed
Source: J Cancer. 2020 May 22;11(16):4709–15. doi: 10.7150/jca.44581 (PMC7330703; doi:10.7150/jca.44581)
Supplement: Supplementary file 1 — Supplementary figures. [file jcav11p4709s1.pdf]

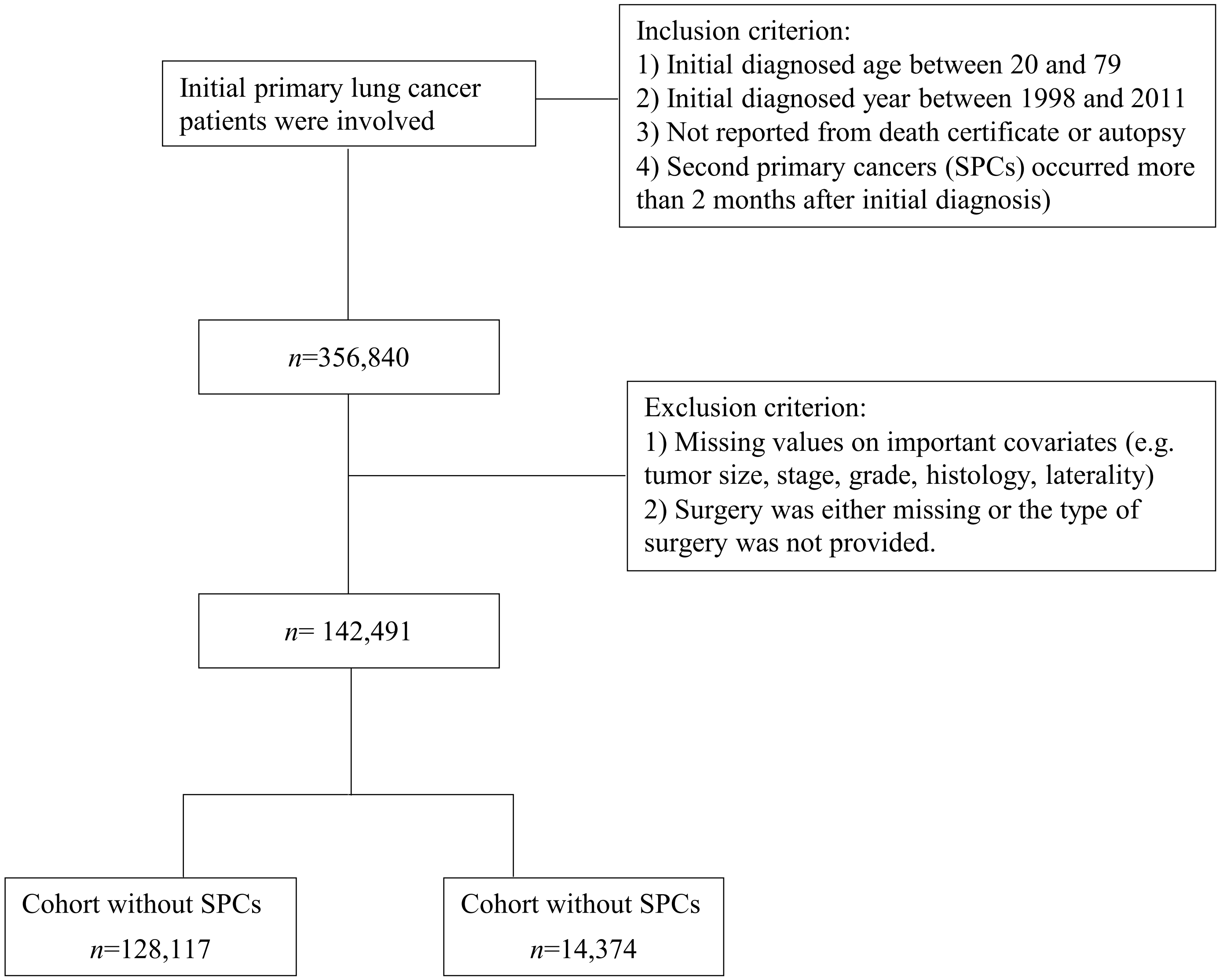

Figure S1 The flowchart of the qualified study patients.

Table S1: Risk scores of each factor listed in the Nomogram

Characteristics	Score
Sex	
Female	0
Male	0.5
Age at diagnosis, years	
20~54	0
55~70	2.8
71~79	1.7
Race	
Black	1.8
White	1.6
Other	0
Tumor size, cm	
<=2	2.3
2~4	1.6
4~6	0.6
>6	0
SEER summary stage	
Localized	9.9
Regional	6.9
Distant	0
Histology	
AD	0.7
LC	1.7
SC	0
SQ	1.5
Other	0.5
Surgery	
No surgery	0
Lobectomy	10
Pneumonectomy	7.8
Sublobectomy	8.6

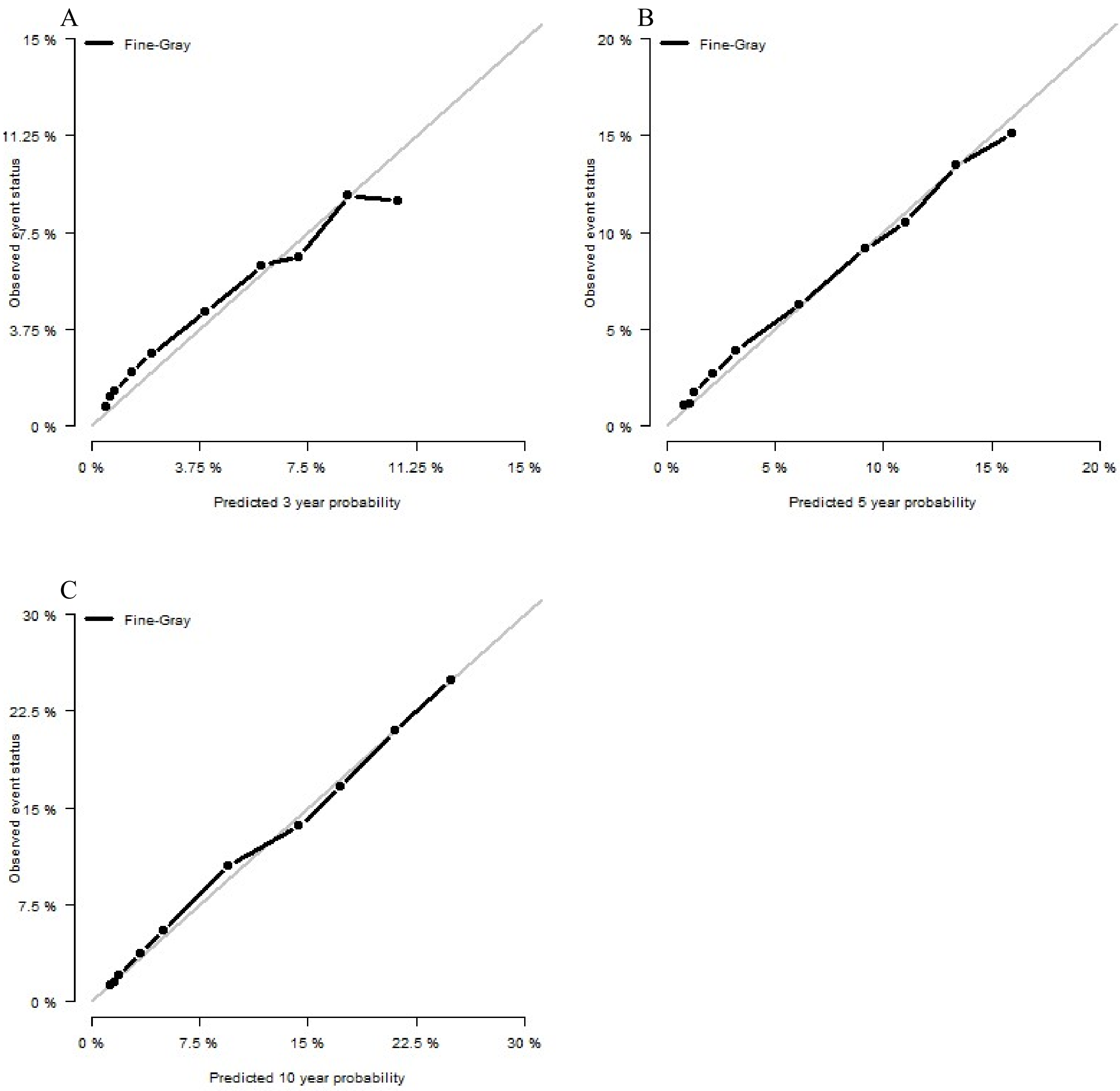

Figure S2. Calibration plots. The x-axis shows the predicted probability of the cumulative incidence model. The y-axis shows the observed cumulative incidences.
